# Vitamin-D receptor agonist calcitriol reduces calcification *in vitro* through selective upregulation of *SLC20A2* but not *SLC20A1* or *XPR1*

**DOI:** 10.1038/srep25802

**Published:** 2016-05-17

**Authors:** M. P. Keasey, R. R. Lemos, T. Hagg, J. R. M. Oliveira

**Affiliations:** 1 Department of Biomedical Sciences – Quillen College of Medicine, East Tennessee State University, Johnson City, USA; 2 Keizo Asami Laboratory – Federal University of Pernambuco, Recife-PE, Brazil; 3 Neuropsychiatry Department – Federal University of Pernambuco, Recife-PE, Brazil

## Abstract

Vitamin D deficiency (hypovitaminosis D) causes osteomalacia and poor long bone mineralization. In apparent contrast, hypovitaminosis D has been reported in patients with primary brain calcifications (“Fahr’s disease”). We evaluated the expression of two phosphate transporters which we have found to be associated with primary brain calcification (*SLC20A2*, whose promoter has a predicted vitamin D receptor binding site, and *XPR1*), and one unassociated (*SLC20A1*), in an *in vitro* model of calcification. Expression of all three genes was significantly decreased in calcifying human bone osteosarcoma (SaOs-2) cells. Further, we confirmed that vitamin D (calcitriol) reduced calcification as measured by Alizarin Red staining. Cells incubated with calcitriol under calcifying conditions specifically maintained expression of the phosphate transporter *SLC20A2* at higher levels relative to controls, by RT-qPCR. Neither *SLC20A1* nor *XPR1* were affected by calcitriol treatment and remained suppressed. Critically, knockdown of *SLC20A2* gene and protein with CRISPR technology in SaOs2 cells significantly ablated vitamin D mediated inhibition of calcification. This study elucidates the mechanistic importance of *SLC20A2* in suppressing the calcification process. It also suggests that vitamin D might be used to regulate *SLC20A2* gene expression, as well as reduce brain calcification which occurs in Fahr’s disease and normal aging.

Vitamin D deficiency is associated with a number of conditions, including osteomalacia and poor long bone mineralization, often found in the elderly and young children (Ricketts). Vitamin D consumption is promoted and also increasingly prescribed in the clinic to avoid osteoporosis. However, the role of vitamin D in soft tissue mineralization is inconsistent. Indeed, excess vitamin D leads to hypercalcaemia and hyperphosphataemia while low or high vitamin D is associated with arterial stiffness and medial calcification^1^. Vitamin D also seems to be involved in gene regulation, e.g., its deficiency leads to increased pro-inflammatory cytokine release in endothelial cells with TNFα capable of inducing osteoblast markers in vascular smooth muscle cells^2^ and vitamin D has been reported to regulate 100’s of genes in smooth muscle cells^3^.

We and others have made incidental reports of hypovitominosis D in patients that display calcifications in the brain^4^,^5^. In addition, vitamin D receptor (VDR) knockout mice exhibit calcifications of the thalamus^6^. These reports suggest that hypovitaminosis D or specific genes vitamin D regulates could be contributing factors in the development of brain calcification. Calcification of the brain is an incidental finding in many patients who undergo brain imaging techniques. As many as 20% of patients over the age of 50 undergoing a CT scan will display mineralization of the brain with no obvious cause or clinical symptoms^7^. Primary familial brain calcification (PFBC; Fahr’s disease; idiopathic basal ganglia calcification) displays an autosomal dominant pattern of inheritance. Typically, PFBC patients show mineralization of the basal ganglia and thalamus with a more intense pattern relative to age-matched controls. The condition is often associated with a diverse array of clinical symptoms including migraine, parkinsonism and dystonia^8^. Several genes have been associated with PFBC, including two phosphate transporters *SLC20A2* and *XPR1*^4^,^5^,^9^,^10^,^11^, and the cytokine *PDGFB* and its cognate receptor PDGFRB^7^,^12^,^13^. Intriguingly, we have also found that mutations in *SLC20A2* represent approximately 50% of the mutations associated with PFBC^14^,^15^. This suggests that *SLC20A2* is the major protagonist in this condition. This view is strengthened by the finding that *SLC20A2* knockout mice present similar calcification patterns as human patients with mutations in the same gene^10^.

*SLC20A2* and *SLC20A1* were initially identified as retroviral transporter proteins^16^,^17^ but have subsequently been shown to function as Na^+^-dependent inorganic phosphate transporters^18^. Both *SLC20A2* (the protein also known as sodium-dependent phosphate transporter 2 or as PiT2) and *SLC20A1* (PiT1) are highly related and maintain ~62% amino acid similarity. However, *SLC20A1* knockout in mice is embryonically lethal due to liver defects^19^. In contrast, *SLC20A2* knockout mice demonstrate no overt effects on development except calcification of the brain^10^. This suggests that *SLC20A2* might have a selective role in mineralization/calcification. Inorganic phosphate (Pi) is critical for bone mineralisation and forms an integral part of hydroxyapatite crystals. The recent reports of mutations in *SLC20A2* associated with PFBC further suggest the importance of phosphate transporters in ectopic calcification^5^,^9^,^20^,^21^.

In the present study, we tested *in vitro* whether vitamin D could suppress calcification, regulate expression of the phosphate transporters associated with PFBC (*SLC20A2* and *XPR1*) and one not associated (*SLC20A1*), and whether these genes would mediate the effects of vitamin D.

## Results

### Calcification is inhibited by chronic treatment with the VDR agonist calcitriol *in vitro*

**Figure 1:**
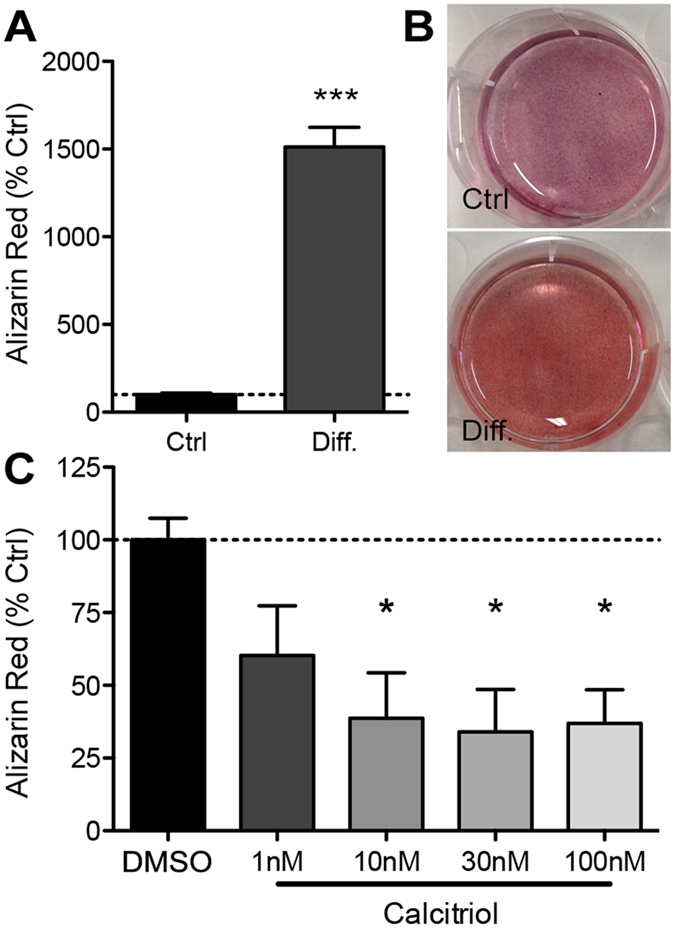
VDR agonist calcitriol reduces calcification *in vitro*. SaOs-2 cells were differentiated (Diff.) with ascorbic acid (AA) and β-glycerophosphate (βG) for 14 days before calcification was quantified by Alizarin Red staining followed by spectrophotometry. (**A**) AA and βG induced significantly more calcification relative to untreated, age-matched controls (***p < 0.001 t-test). (**B**) Intensity of Alizarin Red staining was greater in AA and βG treated cells relative to undifferentiated cells (Ctrl). (**C**) Calcitriol attenuated calcification *in vitro* in a dose dependent fashion (*p < 0.05; one way ANOVA) relative to differentiated (AA + βG) controls treated with vehicle (DMSO). Bars represent means of 3–4 independent experiments ± SEM.

To test the effects of vitamin D, we obtained human SaOs-2 osteoblast-like cells which have previously been shown to undergo differentiation and calcification *in vitro*^23^. SaOs-2 cells were incubated under calcifying conditions using ascorbic acid (AA) and β-glycerophosphate (βG) for 14 days before cells were fixed and calcification confirmed by Alizarin Red staining (Fig. 1A,B). Next, cells were incubated under calcifying conditions with or without the active form of vitamin D, calcitriol. Calcitriol significantly decreased calcification *in vitro* in a dose-dependent fashion (10 nM, 30 nM and 100 nM all *p < 0.05, Fig. 1C) relative to controls treated with vehicle (DMSO).

### SLC20A2, SLC20A1 and XPR1 mRNA expression is reduced by AA/βG-mediated differentiation *in vitro*

**Figure 2:**
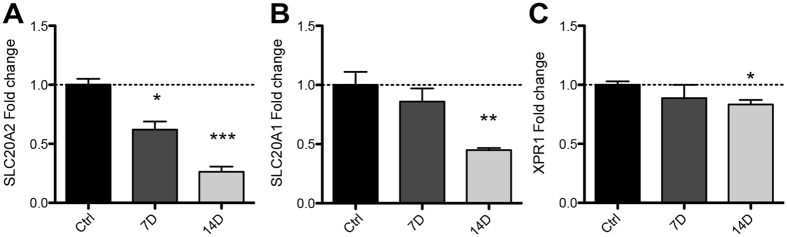
*In vitro* calcification reduces expression of Pi transporters *SLC20A2, SLC20A1* and *XPR1* in SaOs-2 cells. RNA was isolated from SaOs-2 cells treated with AA and βG for 7 or 14 days. (**A**) *SLC20A2* expression was significantly decreased at 7 and 14 days while *SLC20A1* (**B**) and *XPR1* (**C**) mRNA expression was not affected at 7 days, but was significantly decreased at 14 days. Bars represent means of 3 independent experiments ± SEM with one-way ANOVA (p < 0.05*, 0.01**, 0.001***).

Mutations in the phosphate transporters *SLC20A2* and *XPR1* have been implicated in PFBC pathology. *SLC20A1* was included as a control as this phosphate transporter is critical in bone formation^24^. We cultured SaOs-2 cells under calcifying conditions and isolated RNA at 7 or 14 days. Gene expression was quantified by real time quantitative polymerase chain reaction (RT-qPCR). *SLC20A2* mRNA expression was significantly decreased by ~40% (p < 0.05, Fig. 2A) at 7 days and ~75% at 14 days (p < 0.001). *SLC20A1* expression was not affected at 7 days but was decreased 55% at 14 days (p < 0.01, Fig. 2B). XPR1 was not affected at 7 days but was modestly decreased at 14 days (~15%, p < 0.05, Fig. 2C).

### Calcitriol specifically blocked AA/βG-mediated decrease of SLC20A2, but not SLC20A1 or XPR1, in differentiated SaOs-2 cells

**Figure 3:**
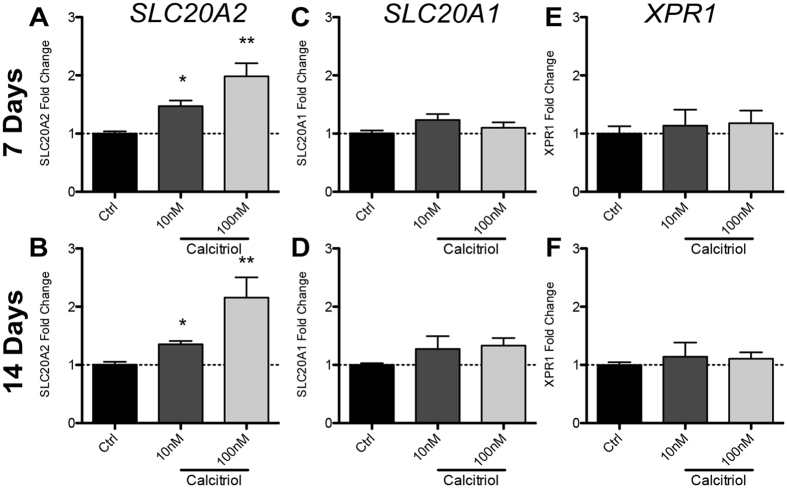
Calcitriol attenuates AA/βG-induced *SLC20A2* mRNA reduction but does not affect *SLC20A1* or *XPR1*. *SLC20A2, SLC20A1* and *XPR1* expression was assessed by RT-qPCR in AA/βG treated SaOs-2 cells co-incubated with calcitriol. (**A**,**B**) *SLC20A2* mRNA expression was significantly higher in cells co-incubated with calcitriol at 7 (**A**) and 14 (**B**) days relative to AA/βG and vehicle treated controls. *SLC20A1* (**C**,**D**) and *XPR1* (**E**,**F**) were not affected it either time point. Bars represent means of 3 independent experiments ± SEM with one-way ANOVA (p < 0.05*, 0.01**).

To test whether the inhibitory effect of calcitriol treatment on calcification (Fig. 1C) could act by modulating *SLC20A2, SLC20A1* or *XPR1*, we performed RNA extractions from differentiated SaOs-2 cells co-incubated with the VDR agonist. Calcitriol was used at a concentration of 10 nM or 100 nM as these concentrations were sufficient to significantly reduce calcification. At 7 days, we found that *SLC20A2* mRNA expression was significantly elevated when cells were incubated with 10 nM (1.5 fold, p < 0.05, Fig. 3A) or 100 nM (2 fold, p < 0.01, Fig. 3A) relative to untreated, differentiated controls (AA/βG). Similarly, *SLC20A2* was upregulated ~1.5 fold and ~2 fold at 14 days for 10 nM and 100 nM calcitriol, respectively (10 nM, p < 0.05; 100 nM, p < 0.01, Fig. 3B) relative to controls. Conversely, neither *SLC20A1* (7 days, Fig. 3C; 14 days Fig. 3D) or *XPR1* (7 days, Fig. 3E; 14 days Fig. 3F) mRNA expression was affected by calcitriol *in vitro*.

### SLC20A2 knockdown by CRISPR ablates inhibition of calcification by calcitriol in SaOs-2 cells

**Figure 4:**
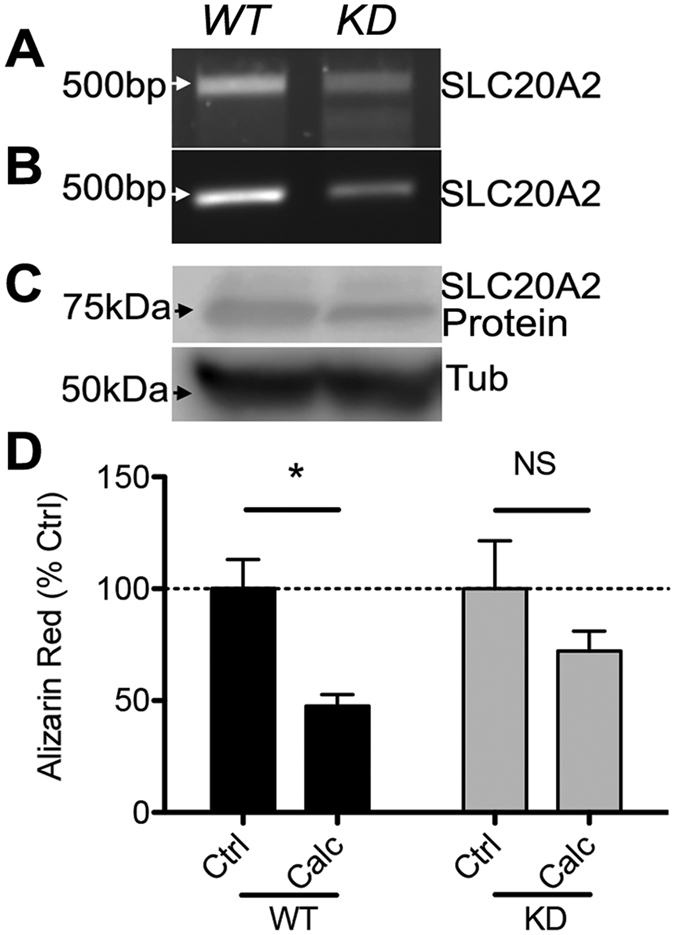
SLC20A2 Knock Down ablates calcitriol-mediated blockade of calcification. SaOs-2 cells were transfected with guide-RNA against SLC20A2 together with Cas-9. (**A**) Gene knockdown was confirmed by PCR amplification of the targeted region and T7 digests to confirm site cutting. Note the reduction in ~500 bp fragments and the appearance of shorter fragments in the CRISPR cells, which is the result of mismatch pairing of the DNA after repair by non-homologous end joining. (**B**) Primers were designed to flank the guide-RNA targeting site and genomic PCR performed on DNA from cells transfected with a donor plasmid (containing a Puromycin resistance gene flanked by homologous arms complimentary to the gene insertion site) with Cas-9. The reduced presence of a ~500 bp fragment is caused by the puromycin insertion, extending the size of the amplicon, thereby producing a diminished band in the PCR reaction. (**C**) Western blot analysis confirmed that protein expression was reduced in CRISPR-treated cells (representative of n = 2). (**D**) Wild type (WT) or SLC20A2 knock down (KD) SaOs-2 cells were treated for 14 days under calcifying conditions with or without calcitriol (Calc). KD cells were no longer protected from calcification by calcitriol (n = 3 independent experiments, *p < 0.05 by two-way ANOVA).

Clustered regularly-interspaced short palindromic repeats (CRISPR) is a native bacterial adaptive immune mechanism which is now being utilized as a novel breakthrough technology to readily perform genetic manipulations. We performed CRISPR-Cas9 mediated knockdown of *SLC20A2* to confirm that calcitriol reduces calcification specifically through maintaining higher levels of the *SLC20A2* protein. SaOs-2 cells were co-transfected with guide-RNAs targeting the *SLC20A2* gene together with a Cas-9 and a donor construct containing the puromycin resistance gene. We chose to delete one allele to secure a knockdown rather than a complete knockout which might have been deleterious. Cells were then selected and used for experiments. We confirmed guide-RNA specificity by T7 endonuclease digest from genomic PCR fragments (Fig. 4A) and by genomic PCR of the region containing the donor insertion (Fig. 4B). Next, we used western blot analysis to confirm that *SLC20A2* protein was decreased in puromycin-selected cells (Fig. 4C). Finally, we tested whether the reduction in calcification afforded by calcitriol could be abolished in *SLC20A2* knockdown SaOs-2 cells. Indeed, we show that *SLC20A2* deficient cells were no longer protected by calcitriol against calcification, as measured by extracted Alizarin Red staining (Fig. 4D).

## Discussion

**Figure 5:**
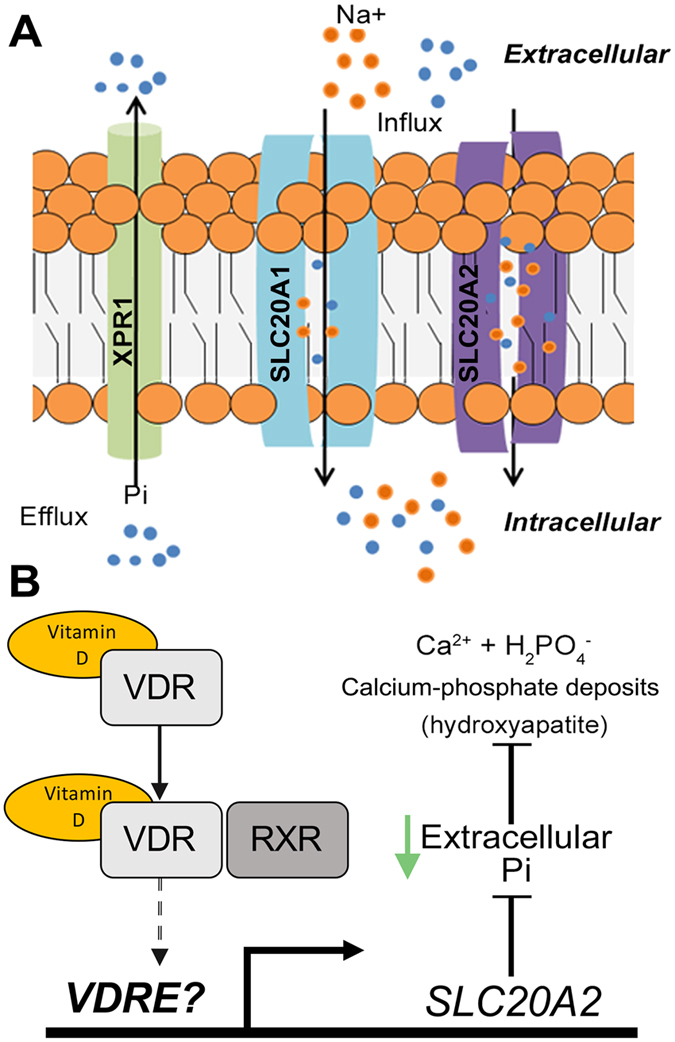
Schematic of Pi transport and proposed model of SLC20A2 function. (**A**) Under physiological conditions, inorganic phosphate (Pi) is taken up by type III sodium-dependent phosphate (NaPi) cotransporters (*SLC20A1* and *SLC20A2*) while Pi can be secreted by *XPR1*. Under pathological conditions, deprivation of vitamin D leads to decreased expression of SLC20A2, leading to Pi imbalances (**B**) Vitamin D binds to the vitamin D receptor triggering translocation to the nucleus, dimerisation with the retinoid X receptor and binding to vitamin D response elements (VDRE) upstream of target genes. Higher expression of SLC20A2 maintains intracellular and reduces extracellular Pi levels thereby inhibiting calcification.

Our current data show that *SLC20A2* mediates the reduction of calcification by cells treated with vitamin D. Mutations for *SLC20A2*^5^,^9^,^14^,^21^ and, more recently, *XPR1*^11^ have been found in PFBC patients, typified by brain calcification. No mutations in *SLC20A1* have been associated with PFBC to date, presumably as loss of this gene has been found to be embryonically lethal^19^. Pi is predominantly found in bones (80% of total body Pi) but 14% is held intracellularly in all cells with the remaining 1% localised in extracellular fluids^34^. *In vitro*, high concentrations of Pi in the medium are sufficient to induce the osteogenic markers osteopontin^35^, RUNX2/Cbfa1 and alkaline phosphatase^36^,^37^. In our study, high extracellular Pi concentration led to significant and robust decreases in *SLC20A2* and *SLC20A1* mRNA expression with a small decrease in *XPR1*. This reduction in Pi transporters suggests a diminished ability of cells to maintain Pi homeostasis (Fig. 5A). The altered gene expression observed in our study could be a consequence of the calcification and the differentiation process. However, that calcitriol can increase *SLC20A2* but not *SLC20A1* or *XPR1* mRNA, and, that knock down of *SLC20A2* prevents calcitriol activity in this model, suggests that calcification occurs after phosphate transporter expression is decreased and Pi homeostasis is de-regulated. We speculate that vitamin D maintains cells in an undifferentiated state via activation of genes such as SLC20A2, thereby maintaining intracellular and extracellular levels of inorganic Pi. Intriguingly, Pi concentration has been reported to alter *SLC20A2* protein oligomeric structure in the cell membrane, independent of its phosphate transport abilities^39^. This may suggest a possible Pi sensing property of the protein. In a separate study, Pi concentration regulated expression of several inhibitors of bone formation, an effect that was abolished by knockdown of *SLC20A1*^38^.

We used a recognised model of *in vitro* calcification, utilising the osteosarcoma SaO-2 cell line, which can be differentiated using AA and βG. We co-incubated SaOs-2 cells during differentiation with calcitriol for 14 days, which led to diminished calcification as measured by Alizarin Red staining. Similarly, others also found that vitamin D could inhibit osteoblast differentiation and calcification *in vitro*^30^. In addition, activators of the VDR inhibit calcification in vascular smooth muscle cells^31^, while VDR knockout mice (mimicking vitamin D loss) results in calcification of the thalamus in 9–14 month old mice^6^. Clinically, vitamin D *in vivo* is critical for calcium absorption and bone strength where hypovitaminosis D is a direct cause of Ricketts and osteomalacia. However, elevated vitamin D can also lead to vascular calcification, increasing the expression of osteoblast transcription factors in vascular smooth muscle cells^32^. These data support a biphasic role for vitamin D in soft tissue calcification^1^,^33^. Clinically, little data exists on the role of vitamin D in soft tissue calcification with the majority of studies focusing on end stage renal disease (ESRD). ESRD patients often display a hardening and calcification of the coronary arteries^40^. It is interesting to note that ESRD is accompanied by hypocalcemia and low calcitriol levels. Further correlative evidence is found when considering the approximately 20% of patients over 50 show cerebral calcifications in CT scans^7^, particularly intriguing when considered in parallel with the finding that low vitamin D is common in residents of care homes for the elderly^41^. However, that many of these individuals also have other co-morbidities makes deciphering the effects of hypovitaminosis D on soft tissue calcification difficult.

We treated cells under calcifying conditions with calcitriol which significantly reduced calcification at the same time as maintaining elevated levels of *SLC20A2* but not *SLC20A1* or *XPR1*. This suggests that *SLC20A2* is a critical regulator of the calcification process. In a study in vascular smooth muscle cells, others found that Pi transport was affected more by *SLC20A2* knockdown relative to *SLC20A1*^42^. Our data support a direct role for vitamin D mediated regulation of *SLC20A2*. In addition to vitamin D’s role in regulating Pi at a cellular level, vitamin D, parathyroid hormone and fibroblast growth factor 23 have been shown to mediate serum Pi through modulation of renal and intestinal Pi absorption as well as bone metabolism^43^. It is possible that these outcomes are mediated through direct regulation of *SLC20A2* expression. To confirm that calcitriol reduced calcification specifically through SLC20A2, we performed knockdown of the SLC20A2 gene in SaOs-2 cells using CRISPR technology. We generated SaOs-2 cells hypomorphic for SLC20A2 expression as measured by genomic PCR and western blotting. SaOs-2 cells exhibiting hypomorphic SLC20A2 expression were no longer protected by calcitriol against the effects of calcification as measured by Alizarin Red. The robust effect of SLC20A2 hypomorphic knockdown suggest that mutations in patients might cause a dysfunctional response to vitamin D rather than a lack of a response.

Vitamin D has been reported to play a number of biological roles with most attention focusing on its importance in bone development but also cancer^25^, cell death^26^ and signalling pathways^27^. Vitamin D is processed into its active form to produce calcitriol, which binds the nuclear vitamin D receptor (VDR)^28^. Activated VDR then interacts with the retinoid X receptor and subsequent binding to vitamin D response elements found in the promoter regions of many genes, thereby regulating transcription^29^. Others found that 176 genes were regulated by calcitriol (115 up and 61 downregulated) in vascular smooth muscle cells *in vitro*^3^. Regulated genes included those involved in cell proliferation, development and ion transporter activity^3^. Here, we showed that the VDR agonist calcitriol induced expression of SLC20a2, but not SLC20a1 or XPR1 in osteoblast-like cells, extending the activity of vitamin D on another important gene and cellular process, as well as confirming its gene-selective effects. We performed *in silico* analysis of the respective promoters of these genes for potential vitamin D response elements using the Contra version 2 website^22^ (data not shown, http://bioit.dmbr.ugent.be contrav2/index.php). Consistent with the expression data, we found possible binding sites within 500 bp upstream of the transcriptional start site for the heterodimer RXR-VDR, suggesting that *SLC20A2* is regulated by the VDR. However, *SLC20A1* contained only a predicted site for VDR but not RXR, while *XPR1* had no predicted VDREs (data not shown). It remains to be determined whether SLC20A2 can bind VDR and RXR. Together with the finding that the SLC20A2 hypomorphic cells were non-responsive to vitamin D with regards to calcification, this data strongly supports the hypothesis that vitamin D abolishes calcification through maintaining increased levels of SLC20A2. This also supports the idea that SLC20A2 Fahr’s disease is caused by mutations that cause hypofunction, and is consistent with the *SLC20A2* knockout mice which show similar calcification patterns^10^.

In conclusion, in line with previous reports, we found that vitamin D can reduce calcification *in vitro*. Calcitriol could reduce calcification *in vitro* while maintaining elevated expression of *SLC20A2* but not *SLC20A1* or *XPR1*. We also found calcitriol blocked calcification directly through the phosphate transporter SLC20A2 (Fig. 5B). This finding adds to the importance of *SLC20A2* in the process of calcification and its role on PFBC. These data may also have implications for regular age-related soft-tissue calcification processes such as seen in the brain, suggesting that sufficient vitamin D intake and uptake should be verified in the ageing population.

## Materials and Methods

### *In vitro* SaOs-2 calcification model

Human SaOs-2 osteoblastic cells were obtained from a public tissue bank at the Federal University of Rio de Janeiro (UFRJ) and maintained in a humidified incubator at 37 °C with 7% CO_2_. This cell line was originally derived from an osteosarcoma obtained from an 11 year old Caucasian female. For experiments, cells were maintained in DMEM (Invitrogen #11960) supplemented with 10% FBS (Invitrogen Cat#16000-044), 1 mM L-glutamine (Invitrogen, Cat#25030) and 50 units/ml Penicillin and 50 μg/ml Streptomycin (Invitrogen, Cat#15140) plated at 10^5 ^cells/ml in 6-well plates. Cells were maintained for 7 days (until reaching ~95%) before 10 mM βG (Sigma, Cat#G9422) and 0.25 mM ascorbic acid (Sigma, Cat#A4544) were added to wells to induce calcification. Media changes and βG addition were performed every 3 days while ascorbic acid was added daily. Cultures were maintained under calcifying conditions for 7 or 14 days before cells were fixed or RNA isolated.

### Alizarin Red staining and quantification

Fixed cells were stained for 10 minutes with Alizarin Red (pH4.5, Sigma, Cat#A5533) then washed 3 × 5 minutes with deionised water. Alizarin Red bound to calcium deposits was brought into solution by incubation with 5% Cetylpyridinium Chloride (Sigma, Cat#C0732) for 10 minutes. The resulting solution was then aliquoted into 96 well plates and read using a spectrophotometer at a 570 nm wavelength (BioRad).

### RNA isolation and Real Time quantitative Polymerase Chain Reaction

Total RNA was isolated using the mirVana^TM^ miRNA Isolation kit (Applied Biosystems #AM1560) according to the manufacturer’s protocol. RNA was quantified using a spectrophotometer (Nanodrop, ThermoFisher). A total of 500 ng RNA was reversed transcribed using the RevertAid H Minus First Strand cDNA Synthesis kit (Thermo Scientific Bio #K1632, USA) according to the manufacturer’s protocol and performed in 20 μL reactions. Real-time reactions were performed as follows: 1 μL complementary DNA (cDNA), 3.5 μL H2O, and 5 μL TaqMan^®^ Gene Expression Master Mix (Applied Biosystems, #4369510, Foster City, CA) with 0.5 μL of 20× TaqMan Gene Expression Assay (GAPDH, #Hs02758991_g1; SLC20A1, #Hs00198840_m1; SLC20A1, #Hs00965587_m1; XPR1, #Hs00173707_m1 from Applied Biosystems) in a 10 μL reaction. Real-time PCR reactions were run in triplicate using a 7500 Fast Real-Time PCR system (Applied Biosystems). Data were analyzed using the ΔΔCt method with values normalized to an endogenous housekeeping gene (GAPDH) and values expressed relative to biological control values.

### Western blotting

Protein isolation was performed according to Keasey *et al*.^44^. Antibodies used were alpha tubulin (1:1000, Cell Signaling, Cat# 2125) and PiT-2 (1:500, Santa Cruz, SC-68419). Signal was developed with a HRP conjugated anti-rabbit secondary antibody and ECL substrate.

### Knockdown of SLC20A2 with CRISPR-Cas9

A CRISPR knock-down kit against human SLC20A2 was purchased from OriGene (USA, Cat# KN206029). Transfections were performed as recommended by the manufacturer with some alterations. Briefly, 2 × 10^5^ SaOs-2 cells were seeded into 6 well plates and maintained for 24 hours. Lipofectamine 3000 (Invitrogen, Cat# L30000) was used at a final concentration of 0.5% together with a total of 5 μg plasmid (2.5 μg gRNA or Scrambled control with 2.5 μg donor) per well. Lipofectamine-DNA complexes were made up in Opti-MEM (Invitrogen, Cat# 31985070). Prior to transfection, medium was replaced with growing medium without penicillin-streptomycin. Cells were maintained for 24–48 hours before cells were returned to growth medium. Transfected cells were sub-cultured 5–7 times before puromycin selection (1 μg/ml, Invitrogen, Cat# A1113803) and confirmation of gene knockdown. Genomic PCR primers were designed against regions of the SLC20A2 gene flanking the guide-RNA target sequence and were: Forward 5′-CGCTGACTGAACACAACCAA-3′ and reverse 5′-CTAACTTCCCCAGCCATGAG-3′ to generate an amplicon of 487 bp in a reaction with 100 ng of genomic DNA extracted from cells with the DNeasy Blood and Tissue kit (Qiagen, USA, Cat# 69506). PCR products were run in an agarose gel for visualization or purification. For T7 digests, PCR bands were excised from agarose using the QIAquick Gel Extraction kit (Qiagen, Cat# 28704). A hybridization reaction was then performed in a PCR cycler with 200 ng of purified PCR product with 2 μl of NEB buffer 2 in a 20 μl reaction. Cycling conditions were 95 °C, 5 min; ramp down to 85 °C (−2 C/s); ramp down to 25 °C (−0.1 C/s); hold at 4 °C. T7 endonuclease (1 U, NEB, CAT# M0302) was added reaction^−1^ and incubated at 37 °C for 15 min. EDTA (2 μl of 0.25 M stock) was added to stop the reaction before samples were electrophoretically separated in an agarose gel.

### Statistics

Data were analysed using GraphPad Prism (Version 5a) software using a One-Way Anova with a Dunnett’s post-hoc test, a student’s t-test or a two-way ANOVA with Bonferroni correction as stated.

## Additional Information

**How to cite this article**: Keasey, M. P. *et al*. Vitamin-D receptor agonist calcitriol reduces calcification *in vitro* through selective upregulation of *SLC20A2* but not *SLC20A1* or *XPR1. Sci. Rep.* **6**, 25802; doi: 10.1038/srep25802 (2016).
